# The Effect of the Anticipated Nuclear Localization Sequence of ‘*Candidatus* Phytoplasma mali’ SAP11-like Protein on Localization of the Protein and Destabilization of TCP Transcription Factor

**DOI:** 10.3390/microorganisms9081756

**Published:** 2021-08-17

**Authors:** Alisa Strohmayer, Timothy Schwarz, Mario Braun, Gabi Krczal, Kajohn Boonrod

**Affiliations:** RLP AgroScience GmbH, AlPlanta Institute for Plant Research, Breitenweg 71, 67435 Neustadt, Germany; alisa.strohmayer@web.de (A.S.); Timothy.Schwarz@dlr.rlp.de (T.S.); Mario.Braun@agroscience.rlp.de (M.B.); gabi.krczal@agroscience.rlp.de (G.K.)

**Keywords:** phytoplasmas, ‘*Candidatus* Phytoplasma mali’, SAP11, plant-pathogen interaction, TCP transcription factors

## Abstract

SAP11 is an effector protein that has been identified in various phytoplasma species. It localizes in the plant nucleus and can bind and destabilize TEOSINE BRANCHES/CYCLOIDEA/PROLIFERATING CELL FACTOR (TCP) transcription factors. Although SAP11 of different phytoplasma species share similar activities, their protein sequences differ greatly. Here, we demonstrate that the SAP11-like protein of ‘*Candidatus* Phytoplasma mali’ (‘*Ca*. P. mali’) strain PM19 localizes into the plant nucleus without requiring the anticipated nuclear localization sequence (NLS). We show that the protein induces crinkled leaves and siliques, and witches’ broom symptoms, in transgenic *Arabidopsis thaliana* (*A. thaliana*) plants and binds to six members of class I and all members of class II TCP transcription factors of *A. thaliana* in yeast two-hybrid assays. We also identified a 17 amino acid stretch previously predicted to be a nuclear localization sequence that is important for the binding of some of the TCPs, which results in a crinkled leaf and silique phenotype in transgenic *A. thaliana*. Moreover, we provide evidence that the SAP11-like protein has a destabilizing effect on some TCPs in vivo.

## 1. Introduction

The delivery of effector proteins and small molecules into the plant host is a common strategy of plant pathogens, including bacteria, fungi, oomycetes, and nematodes, to enhance the hosts’ susceptibility and benefit the pathogen’s infectiousness [1]. The function of these effectors ranges from suppression of the plant immune system to alteration of plant behavior and development [1]. Thus, identifying targets of plant pathogen effectors and revealing plant–microbe interactions facilitate the understanding of the infection mechanisms and, consequently, allows phytoplasma diseases to be controlled.

Phytoplasmas are plant pathogenic bacteria that are transmitted by insect vectors and reside in the phloem of their plant hosts. Phytoplasmas are the causative agent of numerous diseases in plants, including important food crops, leading to heavy damage to the host plant, considerable yield loss, and eventual death of the plant. Phytoplasma has been shown to secrete effector proteins that change plant development and increase phytoplasma fitness [2,3]. In particular, the genome of Aster Yellows phytoplasma strain Witches Broom (AY-WB) [4] was mined thoroughly for potential effector proteins by identifying proteins with a *N*-terminal signal peptide (SP) sequence that are secreted via the Sec-depended pathway [5]. One of these secreted AY-WB proteins (SAP) is SAP11, a small effector protein that has been extensively investigated. SAP11 specifically targets the plant cell nucleus via a nuclear localization sequence (NLS) within the protein and requires a host factor plant importin α [5]. Transgenic *A. thaliana* lines expressing AY-WB SAP11 show severe symptoms, including crinkled leaves, crinkled siliques, stunted growth, and an increase in stem number [6]. The biochemical analysis of transgenic plants expressing AY-WB_SAP11 shows that SAP11 binds and destabilizes CINCINNATA (CIN)-related TEOSINTE BRANCHED1, CYCLOIDEA, PROLIFERATING CELL FACTORS (TCP) transcription factors leading to a decrease of jasmonate (JA) production and an enhanced insect vector reproduction [6,7]. Similar changes in phenotype, in addition to a reduction of JA production and enhancement of insect progeny, can also be found in *A. thaliana* plants infected with AY-WB phytoplasma [7].

In ‘*Candidatus* Phytoplasma mali’ (‘*Ca*. P. mali’) strain AT, a putative pathogenesis-related effector protein ATP_00189 (GenBank: CAP18376.1) was identified that shares 41% of its homology at the amino acid level with AY-WB_SAP11 and was therefore called SAP11-like protein [8]. ‘*Ca*. P. mali’ is the cause of apple proliferation (AP), resulting in symptoms such as witches’ broom, enlarged stipules, and tasteless and dwarf fruits, and thus leading to massive yield losses and economic damage in apple production. AY-WB_SAP11 and the AP_SAP11-like protein share a signal-peptide motif of the phytoplasma-specific sequence-variable mosaic (SVM) protein signal sequence (Pfam entry: PF12113), linking these proteins to a rapid evolution [9]. Both AY-WB_SAP11 and AP_SAP11-like protein are found to be expressed in infected plants [5,7].

SAP11-like protein of ‘*Ca*. P. mali’ STAA, a strain found in Northern Italy, binds TCP transcription factors and the infection with ‘*Ca*. P. mali’ STAA leads to altered phytohormonal levels in apple trees, including changes in JA, salicylic acid, and abscisic acid levels [10]. Furthermore, a change in the odor of the apple tree leads to an enhanced attraction of the insect vector [11,12]. A change in the aroma phenotype, caused by the alteration of volatile organic compound (VOC) production of the plant, is also detected in transgenic *Nicotiana benthamiana* (*N. benthamiana*) plants that express the SAP11-like protein of ‘*Ca*. P. mali’ [13]. The similarities of TCP binding, the biochemical changes in transgenic plants, and hydrophobic amino acid patterns of AY-WB_SAP11 and AP_SAP11-like protein, lead to the assumption that these proteins may have a similar function during phytoplasma infection [10]. Despite the difference in the amino acid feature of the predicted NLS of AP_SAP11-like protein compared to AY-WB_SAP11, the exact function of this domain has not yet been analyzed. Amino acids 40 to 57 of AY-WB_SAP11 have been predicted to function as a bipartite nuclear leading sequence [5]. Disruption of this NLS by deletion of amino acids 56 to 72 or amino acids 40 to 57 leads to a distribution of the protein in the cytoplasm and nucleus [5]. The same effect was reported when only two lysines at position 55 and 56 of the protein were deleted [7]. In contrast, Wang and co-workers [14] showed that the NLS deletion within SWPI, the SAP11-like effector of wheat blue dwarf phytoplasma, did not change the localization pattern of this protein, which is in contrast to the result with AY-WB SAP11. However, transgenic plants expressing either AY-WB SAP11-NLS or its *N*-termini, including NLS deleted mutants, exhibited reduced crinkled leaves and siliques phenotypes [7]. Thus, we question whether the anticipated NLS of AP_SAP11-like_PM19 has the similar functions.

The aim of this study was to analyze a stretch of ‘*Ca*. P. mali’ PM19, a strain found in Southwest Germany that corresponds to the predicted NLS of AY-WB_Sap11 [5], regarding its effect on the localization of SAP11 within the plant cell and the binding and destabilizing of AtTCP transcription factors. Furthermore, we compare our findings with the current knowledge on SAP11 effectors and discuss its possible functions.

## 2. Materials and Methods

### 2.1. Origin of AP_SAP11-Like_PM19 DNA

‘*Ca*. P. mali’ strain PM19 was previously transmitted from field-collected *Cacopsylla picta* to healthy test plants of *Malus* × *domestica* [15]. Total DNA was extracted from plant tissue using a modified cetyltrimethylammonium bromide (CTAB)-based protocol described elsewhere [15]. The gene *AP_SAP11-like_PM19* (GenBank Accession number MK966431) without SMV signal was amplified using primers that were designed based on its corresponding gene of ‘*Ca*. P. mali’ strain AT [16]: 5′-TCTCCTCCTAAAAAAGATTC-3′ and 5′-TTTTTTTCCTTTGTCTTTATTGTT-3′.

### 2.2. Transient Protein Expression in Planta

The *AP_SAP11-like_PM19* gene was codon optimized for expression in *A. thaliana* and synthesized (GeneCust, Ellange, Luxembourg). This version of the gene was used as the basis for all constructs used in in planta experiments in this work.

Agroinfiltration was performed as previously described [17]. A single colony of *Agrobacterium tumefaciens* (*A. tumefaciens*) strain ATHV transformed with pPZP200 binary vector [18] containing the respective gene under control of the *Cauliflower mosaic virus* 35S promoter was grown at 28 °C. Bacteria were centrifuged, resuspended in induction media (bacterial growth medium substituted with 10 mM MES, 2 mM MgSO_4_, and 0.05 M acetosyringone), and grown overnight at 28 °C. After centrifuging, the cell pellet was resuspended to an OD600 of 2.4 in infection media (1/2 MS media, 10 mM MES, 2% sucrose, and 0.2 mM acetosyringone) and incubated at room temperature for at least 2 h. The bacteria were infiltrated into *N. benthamiana* leaves using a 1 mL needleless syringe.

For protoplast isolation, two leaves were collected 2 days after infiltration, cut into small strips of about 1 mm, and placed in enzyme solution for cell wall maceration (1/2 MS, 0.4 M sucrose, 1% Cellulase Onozuka R-10, and 0.2% Macerozyme R-10 (both Serva, Heidelberg, Germany), pH = 5.8). After infiltration by application of vacuum, leaves were incubated for 2 h on a gyratory shaker (20 rpm). Protoplasts were collected by centrifuging at 100× *g* and examined using a Zeiss Observer Z1 with an LSM510 confocal laser-scanning head.

### 2.3. Generation of Transgenic A. thaliana Lines

Floral dip was performed as previously described [19]. A single colony of *A. tumefaciens* strain GV3101 transformed with pPZP200 binary vector [18] containing the respective gene under control of the *Cauliflower mosaic virus* 35S promoter was grown overnight at 28 °C. Bacteria were centrifuged, resuspended in infection media (1/2 MS, 10 mM MES; 5% sucrose, 0.2 mM acetosyringone, and 0.05% Silwet L-77), and incubated at room temperature for at least 2 h. Flowers of *A. thaliana* plants were submerged in the bacteria suspension for 30 s. Plants were kept in a dark and humid area for 2 days and then grown in greenhouse in long-day (16 h/8 h light/dark) conditions until seeds could be collected. F1 and F2 generations of transgenic plants were selected by spraying of BASTA solution, diluted 1/1000 in H_2_O. The F2 generation of transgenic *A. thaliana* lines was screened for phenotypic symptoms and analyzed using RT-qPCR.

### 2.4. RT-qPCR

For RT-qPCR, RNA of transgenic *A. thaliana* plants was extracted using the Macherey-Nagel RNA extraction kit (Macherey-Nagel, Düren, Germany). The resulting RNA was additionally treated with DNase I (according to kit protocol) and tested for DNA contamination by performing RT-qPCR with 10 and 50 ng RNA per reaction. Only samples that did not show any signal in both reactions were used for cDNA synthesis. cDNA was synthesized from 0.5 µg of total RNA using a RevertAid Premium Kit (Fermentas) and subjected to RT-qPCR using iTaq Universal SYBR Green Supermix (BioRad) and 10 ng of cDNA per reaction. RT-qPCR reaction was performed in a Chromo 4TM system cycler (Biorad). Four technical replicates were produced per plant by repeating the cDNA synthesis step separately twice and performing two RT-qPCR reactions per cDNA sample. Two reference genes were used: glyceraldehyde-3-phosphate dehydrogenase (*GAPDH*) and protein phosphatase 2 (*PP2A*). *GAPDH* is a standard reference gene, often used in RT-qPCR [20]. *PP2A* is one of many genes suggested as new reference genes by Czechowski and coworkers due to their stable expression [20]. For *GAPDH* and *PP2A*, primers were used as described [20]. For amplification of *AP_SAP11-like_PM19* and its mutations, gene-specific primers that can bind to all three variants were designed (Supplementary Materials, Table S1). The mean Cq values of the technical replicates, in addition to the standard error of average Cq values, the maximal coefficient variation of Cq within replicate samples, and the average efficiency of genes, were calculated using the Real-Time PCR Miner software [21] and are provided in Supplementary Materials, Tables S2 and S3, respectively. The relative expression levels were calculated using the ddCt method [22] and normalized to the geometric average of the Cq of the reference genes. In the calculation, the relative expression level of each gene was also normalized to that of *AP_SAP11-like_PM19*. The exact equation used for the calculation was as follows:

$$\mathrm{Relative}\;\mathrm{Expression}\;\mathrm{of}\;\mathrm{GOI}\;=\;\frac{E(SAP11{)}^{\mathrm{Cq}(SAP11)-\mathrm{Cq}(\mathrm{GOI})}}{[E(GAPDH{]}^{\mathrm{Cq}(SAP11)-\mathrm{Cq}(GAPDH)}+E(PP2A{)}^{\mathrm{Cq}(SAP11)-\mathrm{Cq}(PP2A)}]/2}$$

### 2.5. Yeast Two-Hybrid Analysis

Y2H screening was performed using the Matchmaker Gold Yeast Two-Hybrid System (Takara Bio USA, Inc., Mountain View, CA, USA). For bait, *AP_SAP11-like_PM19* or *AP_SAP11-like_PM19Δ40-56* was cloned into pGBKT7 (TRP1 nutritional marker) for fusion with the Gal4 binding domain (BD). An empty pGBKT7 vector expressing only BD was used for a negative control. For prey, DNA samples with the genes of all 24 AtTCP transcription factors were purchased from The Arabidopsis Information Resource (TAIR, Phoenix Bioinformatics, Fremont, CA, USA), PCR amplified, and cloned into pGADT7 (LEU2 nutritional marker) for fusion with the Gal3 activation domain (AD). Different yeast drop-out media were purchased from Sigma-Aldrich Inc. (St. Louis, MO, USA) and prepared according to the manufacturer’s instructions. A quantity of 40 µg/mL of X-α-Gal (Iris Biotech GmbH, Marktredwitz, Germany) and 200 ng/mL of AbA (Takara Bio USA, Inc., Mountain View, CA, USA) were added when they were required. Two reporter genes were used to detect protein interaction: *AUR1-C* confers strong resistance to Aureobasidin A (Aba); *MEL1* encodes α-Galactosidase. When the substrate X-α-Gal is added to the media, positive colonies turn blue. To test for interaction between bait and prey, the respective expression plasmids were co-transformed into yeast strain Y2H gold and patched on –Leu/Trp double drop out plates (DDO) that select for the presence of both expression plasmids and DDO plates substituted with X-α-Gal and Aba (DDOXA) to select for positive interactions.

### 2.6. TCP Destabilization Assay

The gene-encoded *AP_SAP11-like_PM19* and *AP_SAP11-like_PM19Δ40-56* were synthesized and subcloned into the binary vector pPZP2000 fused to *GFP*, resulting in pPZP2000- *AP_SAP11-like_PM19-GFP* and *AP_SAP11-like_PM19Δ40-56-GFP*, respectively. The AtTCPs were amplified from the AtTCP collection of The Arabidopsis Information Resource (TAIR, Phoenix Bioinformatics, Fremont, USA-CA). The TCP12 was kindly provided by Prof. Jun-Yi Yang. The DNA fragments were subcloned and fused with HA-tag by the substitute PPT gene, a herbicide resistant gene for transgene plant selection in the pPZP2000- *AP_SAP11-like_PM19-GFP* and *AP_SAP11-like_PM19Δ40-56-GFP* plasmids, resulting in pPZP2000- *AP_SAP11-like_PM19-GFP/HA-AtTCPs* and *AP_SAP11-like_PM19Δ40-56-GFP/HA-AtTCPs*, respectively. Thus, both genes (*SAP11* and *AtTCPs*) were expressed from the same vector under control of double 35S and NOS promoters, respectively. The plasmids were transformed into *A. tumefaciens* strain ATHV. After 2 days, the infiltrated leave were collected and extracted as described by Chang et al. [23]. After SDS-PAGE gel electrophoresis, the specific antibodies anti-GFP (antibody online) and anti-HA (Sigma) were used to monitor the protein amount. All experiments were repeated 3 time using biologically distinct samples.

## 3. Results

### 3.1. Sequence Analysis of SAP11-like Protein of ‘Candidatus Phytoplasma mali’ Strain PM19

**Figure 1:**
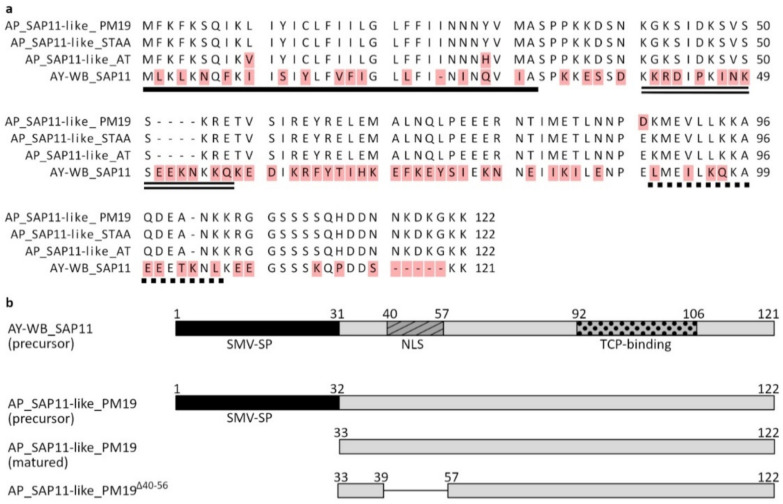
Sequence alignment and schematic representation of the AP_SAP11-like_PM19 amino acid sequence used in this study. (**a**) Multiple sequence alignment of AP_SAP11-like protein of strains PM19, STAA and AT, and AY-WB_SAP11. Annotations: single black line represents sequence-variable mosaic protein signal peptide (SVM-SP), double black line represents nuclear leader sequence (NLS), and the dotted line represents TCP-binding area of AY-WB_SAP11. (**b**) Schematic representation of deletions and additions to AP_SAP11-like_PM19 in comparison to AY-WB_SAP11. The black squares represent the SVM-SP, the striated squares represent the NLS, and the dotted squares represent the TCP-binding area. The deletion of amino acids 40 to 56 in AP_SAP11-like_PM19Δ40–56 is indicated by a line connecting the squares.

The gene of the SAP11-like protein (GenBank Accession number MK966431) studied in this work was isolated from ‘*Ca*. P. mali’ strain PM19 [15]. Its gene product is a 14 kDa protein composed of 122 amino acids. The alignment results of AP_SAP11-like_PM19 show an identity of 40% with AY-WB_SAP11 (Accession Number WP_011412651) and a high similarity to AP_SAP11-like proteins of other strains (Figure 1a). Compared to the STAA [10] and AT [16] strains, there are only one and two amino acids substituted, respectively (Figure 1a).

AY-WB_SAP11 and AP_SAP11-like_PM19 protein share a SVM signal sequence that is located in the *N*-terminal part of the proteins from amino acid 1 to 31 or 32, respectively, and is important for the secretion of the proteins into the plant host cell via the phytoplasma Sec-dependent pathway [5,8]. The secretion of proteins via the Sec-dependent pathway is connected with the cleavage of the signal peptide, resulting in the mature protein [24]. Thus, a truncated version of AP_SAP11-like protein of strain PM19 starting with amino acid 32, called AP_SAP11-like_PM19, was used in all experiments (Figure 1b), unless otherwise indicated.

For AY-WB_SAP11, two more domains were identified: a NLS (amino acids 40 to 57) that is required for nuclear localization, and a TCP-binding area (amino acids 92 to 106) that is part of a predicted coiled coil structure (amino acids 90 to 110) [5,7]. It has been previously discussed that AP_SAP11-like protein and AY-WB_SAP11 share similar hydrophobicity motifs that can be responsible for similar functions of the proteins [10]. However, this has not yet been experimentally determined.

### 3.2. Amino Acids 40 to 56 of AP_SAP11-like_PM19 Are Not Necessary for Nuclear Localization of the Protein in N. benthamiana

**Figure 2:**
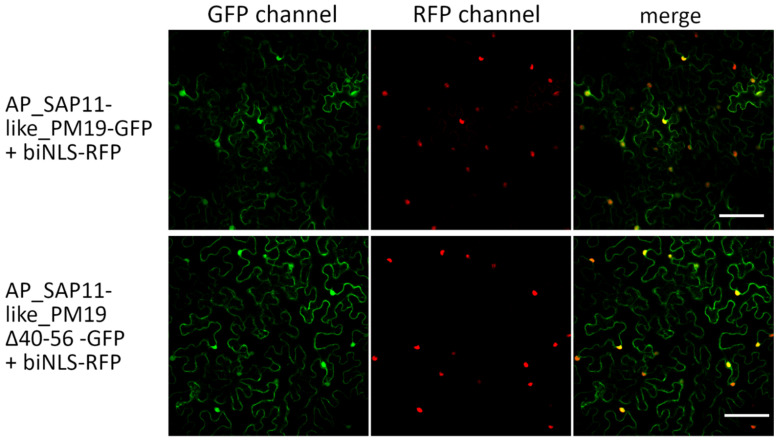
Localization studies of AP_SAP11-like_PM19 protein in infiltrated *N. benthamiana* leaves. AP_SAP11-like_PM19 and AP_SAP11-like_PM19Δ40–56 were fused to GFP and biNLS with RFP to decorate the nucleus. The localization of expressed proteins in infiltrated leaves was analyzed by confocal microscopy using GFP and RFP filters. AP_SAP11-like_PM19 (SAP11-GFP) and AP_SAP11-like_PM19Δ40–56 (ΔNLS-SAP11-GFP) localize not only in the plant nucleus but also in the cytoplasm. Co-localization with biNLS-RFP is indicated by yellow coloring in the merge. Scale bar = 100 µm.

**Figure 3:**
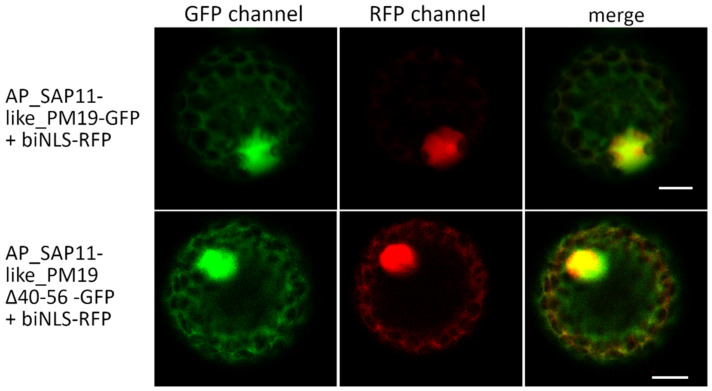
Localization studies of AP_SAP11-like_PM19 protein in protoplasts of infiltrated *N. benthamiana* leaves. AP_SAP11-like_PM19 and AP_SAP11-like_PM19Δ40–56 were fused to GFP and biNLS with RFP to decorate the nucleus. The localization of expressed proteins was analyzed by confocal microscopy using GFP and RFP filters. AP_SAP11-like_PM19 (SAP11-GFP) and AP_SAP11-like_PM19Δ40–56 (ΔNLS-SAP11-GFP) localize not only in the plant nucleus but also in the cytoplasm. Co-localization with biNLS-RFP is indicated by yellow coloring in the merge. Scale bar = 10 µm.

SAP11 of AY-WB phytoplasma was shown to localize in the plant cell nucleus [5]. To elucidate whether SAP11-like protein of AP phytoplasma strain PM19 also locates in the plant cell nucleus, the gene AP_SAP11-like_PM19 was codon-optimized for expression in *A. thaliana*, synthesized (GeneCust, Ellange, Luxembourg), and fused to green fluorescence protein (GFP), resulting in AP_SAP11-like-PM19-GFP. To mark the nucleus of the plants cells, a bipartite nuclear leading sequence (biNLS) of the *Nicotiana tabacum* domains rearranged methyltransferase 1 (NtDRM1) [25] was fused to red fluorescence protein (RFP). The biNLS-RFP was transiently co-expressed with AP_SAP11-like_PM19-GFP in *N. benthamiana* using an Agrobacterium-mediated expression system. Two days after Agrobacterium infiltration, protoplasts were isolated from the infiltrated leaves and analyzed by confocal microscopy using GFP and RFP filters. The results show that the AP_SAP11-like_PM19-GFP is mainly localized in the nucleus marked by biNLS-RFP (Figure 2 and Figure 3).

For AP_SAP11-like_PM19, we were not able to predict any NLS sequence using different software [26,27,28]. Research undertaken to date indicates that AY-WB_SAP11 and AP_SAP11-like protein have a similar function [6,10,13], and thus might also have a similar structure. We hypothesized that a potential NLS of AP_SAP11-like protein may be in a region similar to that of AY-WB_Sap11 NLS. To elucidate whether the area of AP_SAP11-like_PM19 corresponding to the predicted NLS of AY-WB SAP11 in sequence alignment (amino acids 40 to 56, Figure 1a) is necessary for the nuclear localization of the protein, we constructed a truncated version of AP_SAP11-like_PM19 excluding amino acids 40 to 56 (Figure 1b), which correspond to the predicted NLS of AY-WB_Sap11 (Figure 1a), and fused the truncated protein to GFP, resulting in AP_SAP11-like_PM19Δ40–56-GFP. Confocal microscopic analysis of the mesophyll (Figure 2) and the protoplasts isolated from the infiltrated leaves (Figure 3) shows that AP_SAP11-like_PM19Δ40–56-GFP still localizes in the plant cell nucleus. This suggests that the 40–56 amino acid stretch of AP-SAP11-like_PM19 is not required for transporting the protein into the plant cell nucleus.

### 3.3. AP_SAP11-like_PM19 Induces Crinkled Leaves and Siliques and Witches’ Broom Symptoms in Arabidopsis

The recombinant expression of AY-WB_SAP11 in *A. thaliana* induces stem proliferation, leading to witches’ broom symptoms, and alteration of leaf and silique shape [6], and the expression of AP_SAP11-like protein of ‘*Ca*. P. mali’ in *N. benthamiana* also leads to morphological changes with stunted growth and crinkled leaves [13].

**Figure 4:**
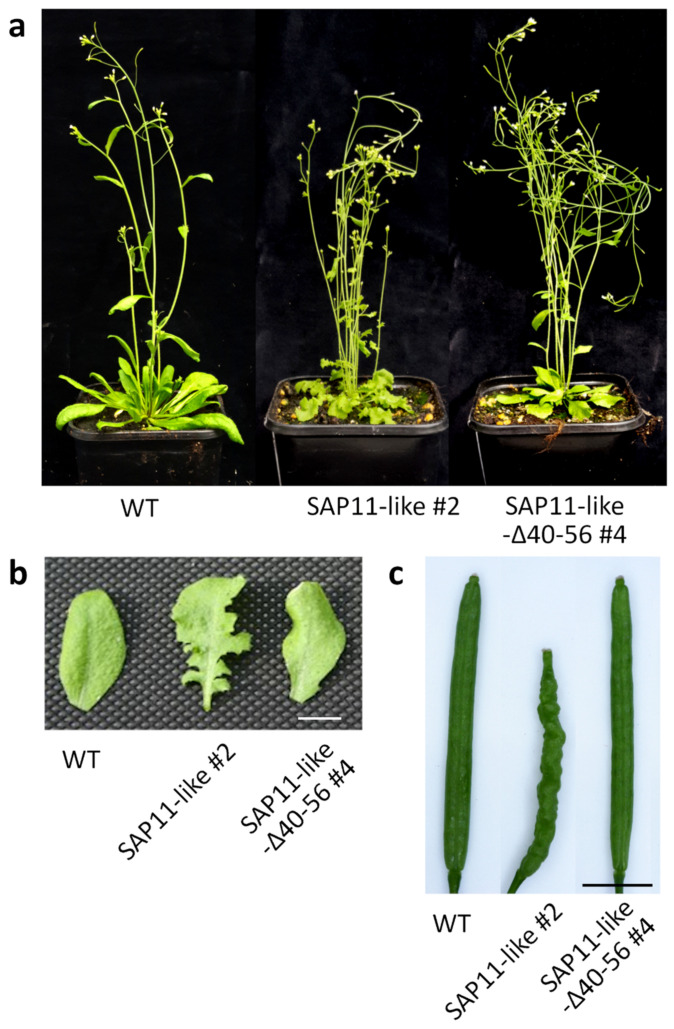
Amino acids 40 to 56 of AP_SAP11-like_PM19 protein are required for symptom development in *A. thaliana*. (**a**) Transgenic *A. thaliana* lines expressing AP_SAP11-like_PM19 (SAP11-like) and AP_SAP11-like_PM19Δ40–56 (SAP11-like-Δ40–56) under the control of the *Cauliflower mosaic virus* 35S promoter compared to the wt Arabidopsis Col-0 plant. All plants were grown for 8 weeks in long-day (16 h/8 h light/dark) conditions. For each of the three constructs, at least 3 lines were examined, all showing similar phenotypic characteristics. (**b**) Leaves of transgenic plant lines shown in (**a**). Scale bar = 1 cm. (**c**) Siliques of transgenic lines shown in (**a**).

**Figure 5:**
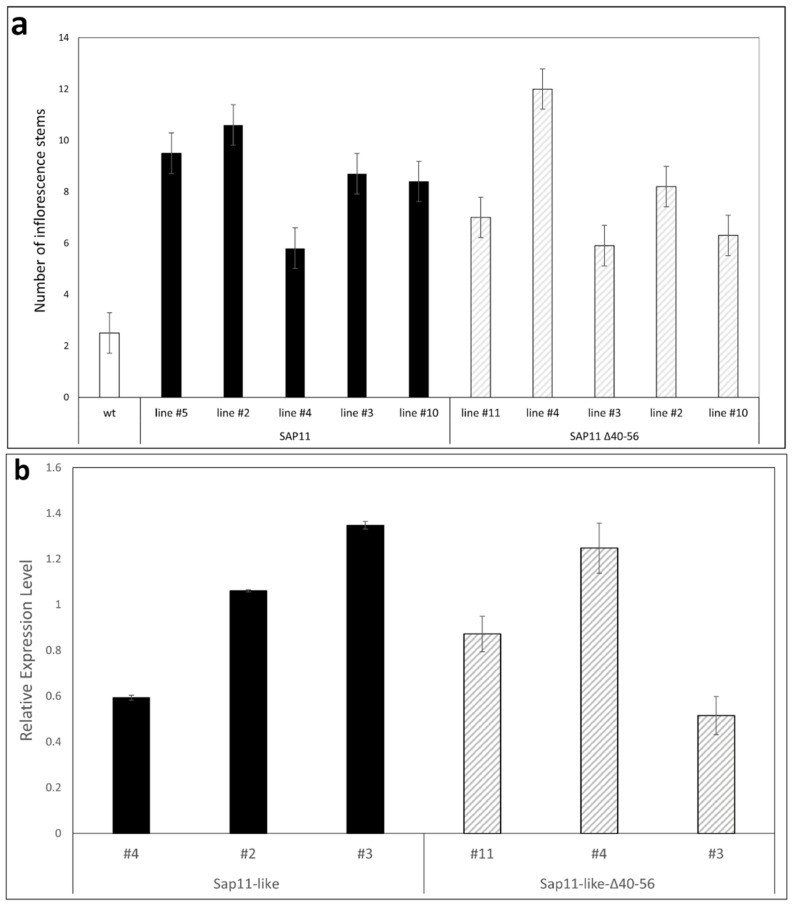
(**a**) Number of primary stems developed by transgenic *A. thaliana* lines compared to the wt Col-0 plant. (**b**) Relative expression levels of transgenes of transgenic lines compared to the geometric average of glyceraldehyde-3-phosphate dehydrogenase (*GAPDH*) and protein phosphatase 2 (*PP2A*). All values show a *p*-value lower than 0.05 in ANOVA.

To analyze the effects of AP_SAP11-like_PM19 protein in *A. thaliana*, we produced transgenic Arabidopsis plant lines stably expressing AP_SAP11-like_PM19 (Figure 1b) under the control of the *Cauliflower mosaic virus* 35S promoter. The transgenic *A. thaliana* plant lines show smaller rosettes and a witches’ broom phenotype (Figure 4a), crinkled leaves (Figure 4b), and crinkled siliques (Figure 4c), in addition to an increase in the number of primary stems (Figure 5a).

### 3.4. Amino Acids 40 to 56 of AP_ SAP11-like Protein Are Important for Symptom Development

It was shown that transgenic *A. thaliana* lines expressing AY-WB_SAP11 missing the *N*-terminal region, including the NLS or an AY-WB_SAP11 mutant lacking two lysines at position 55 and 56, loose the crinkled leave symptom [7]. However, it was not finally determined whether the missing phenotype is caused by the lacking transport of the protein into the nucleus or by the mutation of the protein itself [7]. To reveal the role of the 40 to 56 amino acid region of AP_SAP11-like_PM19 and to address the question of whether the region is important for the symptom development, we produced transgenic Arabidopsis lines expressing AP_SAP11-like_PM19Δ40–56 (Figure 1b).

The transgenic lines expressing AP_SAP11-like_PM19Δ40–56 showed a less severe phenotype compared to the plants expressing AP_SAP11-like_PM19, with a smaller rosette and increase in stem number (Figure 4a and Figure 5a), but the leaves and siliques resembled the wild type (wt) *A. thaliana* plant (Figure 4b,c), suggesting that the 40–56 amino acid stretch of AP_SAP11-like_PM19 is somehow important for symptom development in *A. thaliana*, particularly concerning the morphological changes of leaves and siliques. Similar results were reported for AY-WB_SAP11 [7].

To exclude the effect of the expression level of the transgene in the transgenic plant lines on the phenotype development, we performed RT-qPCR of homozygote transgenic plant lines using AP_SAP11-like_PM19 specific primers that are able to bind both gene constructs, to ensure similar amplification. For each construct, three plant lines were analyzed.

The maximal standard error of average Cq values of all three genes analyzed (*AP_SAP11-like_PM19* and reference genes *GAPDH* and *PP2A* [20]) was 3.6%, and the maximal coefficient variation of Cq within the replicate samples was 7.1%. The average efficiency of all three genes varied between 1.69 and 1.80. For a list of all obtained data see Table S2.

The obtained values were normalized to the arithmetic average of *AP_SAP11-like_PM19* for better comparison. The result in Figure 5b shows that the relative expression level of *AP_SAP11-like_PM19Δ40–56* and the wt *AP_SAP11-like_PM19* are not significantly different. Thus, the result indicates that the different phenotypic developments are not caused by different gene expression levels.

We thus ruled out that the gene expression level is the cause of losing the symptoms of crinkled siliques and leaves in the transgenic lines expressing the Δ40–56 mutant.

### 3.5. Amino Acids 40 to 56 of AP_SAP11-like_PM19 Are Important for Binding to Some A. thaliana (At) TCPs in Yeast Two-Hybrid (Y2H) Analysis

Sugio and co-workers showed that AY-WB_SAP11 interacted with class II AtTCP2 and 13 in Y2H screenings, and with AtTCP2 and 4 of class II and AtTCP7 of class I in co-immunoprecipitation assays [6]. Moreover, a transient co-expression analysis showed that AY-WB_SAP11 destabilizes all class II AtTCP of the CIN-group, but not the class I AtTCP7 [6]. Deletion of the *N*-terminal region of AY-WB_SAP11 including the NLS disturbs its ability to destabilize AtTCP-transcription factors in a co-immunoprecipitation assay [7].

AP_SAP11-like protein of strain STAA interacts with three TCP transcription factors of M. domestica (MdTCP) that are homologous to members of AtTCP class II, namely, MdTCP25 (a homolog of AtTCP4), MdTCP24, (a homolog of AtTCP13), and MdTCP16 (an isoform of AtTCP18) [10]. In planta interaction of AP_SAP11-like protein strain STAA with MdTCP24 and 25 was confirmed using bimolecular fluorescence complementation (BiFC) [10]. To the best of our knowledge, to date, no interaction of AP_SAP11-like protein with members of AtTCP class I has been reported.

**Figure 6:**
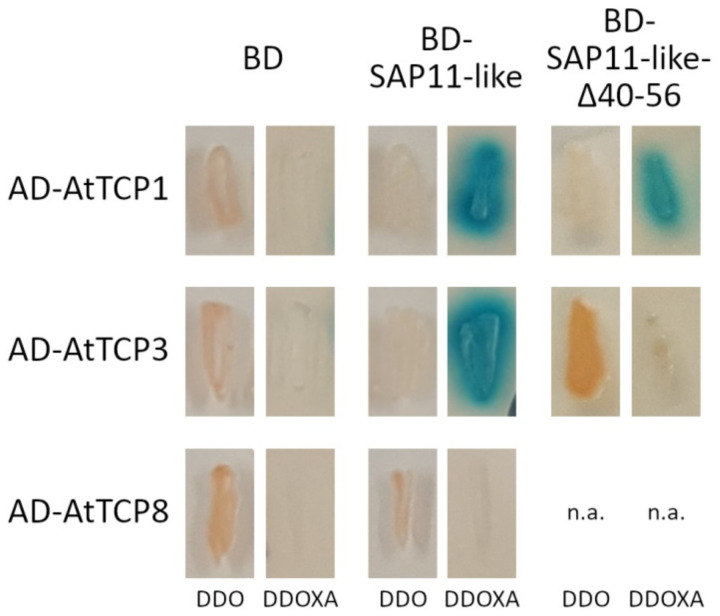
Example for Y2H results of AP_SAP11-like_PM19 and AP_SAP11-like_PM19Δ40–56 with different AtTCPs. Y2H screens were performed using the binding domain fused to AP_SAP11-like_PM19 (BD-SAP11-like) or AP_SAP11-like_PM19Δ40–56 (BD-SAP11-like-Δ40–56) and the activation domain (AD) fused to different AtTCPs. For the negative control, only BD was used. Co-transformed yeast cells were patched on double drop-out medium (DDO) to select for presence of both expression plasmids and DDO media containing Aureobasidin A and X-α-Gal (DDOXA) to select for protein interaction. n.a. = not applicable. A figure showing all Y2H results can be found in the Supplementary Materials (Figure S1).

Because our results indicate that the 40–56 amino acid stretch of AP_SAP11-like_PM19 is not responsible for nuclear localization of the protein, but responsible for some of the phenotypic characteristics of the transgenic Arabidopsis expressing AP_SAP11-like protein, we further analyzed its possible role during the interaction with AtTCP transcription factors using Y2H screens. In the first step, we screened for interaction of AP_SAP11-like_PM19 with all AtTCP transcription factors identified thus far [29]. We used the AP_SAP11-like protein fused to the Gal4 binding domain (BD) for bait (expression plasmid pGBKT7-*AP_SAP11-like_PM19*) and the AtTCP transcription factors fused to the Gal4 activation domain (AD) for prey (expression plasmid pGADT7-*AtTCP*). The pGBKT7-empty plasmid expressing only BD was included as the negative control. Co-transformed yeast cells carrying the bait or pGBKT7, and one of the candidate prey plasmids, were screened for resistance to Aureobasidin A (AbA) and expression of α galactosidase. All Y2H results are given in the Supplementary Materials (Figure S1), and examples for positive and negative Y2H results are shown in Figure 6. All Y2H results are summarized in Table 1.

All eleven members of the AtTCPs class II (AtTCP1-5, 10, 12, 13, 17, 18, and 24) and six of thirteen members of AtTCPs class I (AtTCP6, 7, 9, 14, 19, and 21) showed activation of both reporter genes in combination with AP_SAP11-like_PM19, whereas the remaining seven AtTCPs of class I (AtTCP8, 11, 15, 16, 20, 22, and 23) and all negative controls did not. This result indicates that AP_SAP11-like_PM19 protein does not only exclusively interact with class II but also with some of the class I AtTCPs.

In the next step, we repeated Y2H screens of all candidate prey that showed interaction with AP_SAP11-like_PM19, using the AP_SAP11-like_PM19Δ40–56 deletion mutant for bait. The result shows that AP_SAP11-like_PM19Δ40–56 binds to all tested AtTCPs, with the exception of AtTCP19 of class I, and AtTCP3, 4, and 10 of class II. The results clearly indicate that the 40–56 amino acid stretch is important for the interaction with some AtTCPs, particularly those of class II.

### 3.6. The 40–56 Amino Acid Stretch of AP_SAP11-like_PM19 Is Not Required to Destabilize Some of the TCPs

**Figure 7:**
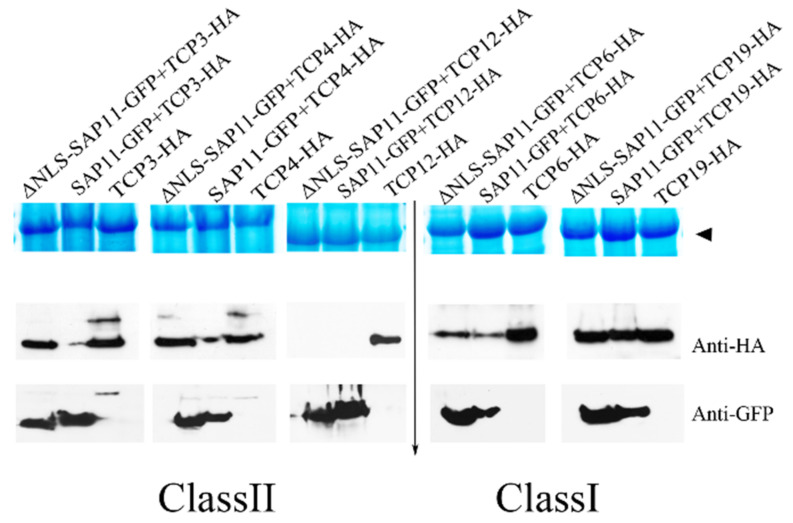
TCP destabilization assay. The relative quantities of AtTCPs (3, 4, 6, 12, and 19) were monitored in the presence of AP_SAP11-like_PM19 and AP_SAP11-like_PM19Δ40–56 when they were transiently expressed from the same vector in *N. benthamiana*. The amounts of AtTCPs and SAP11 were examined using a monoclonal anti-HA for detecting HA-AtTCPs (middle panel) and anti-GFP for SAP11-GFP (**low panel**) in Western blotting analysis. For controlling the total protein loading amount, the large subunit of Rubisco visualized with Coomassie Brillant Blue staining is indicated by an arrowhead (**upper panel**).

It was shown that SAP11 binds and destabilizes AtTCPs in vivo [7]. Although the Y2H results showed that AP_SAP11-like_PM19Δ40–56 binds to some of the TCPs (with the exception of TCP3, 4, 10, and 19), and the wt protein, this does not necessarily translate into the TCP destabilization. Therefore, we further investigated its destabilizing activity compared to the wt protein. We adapted the agroinfiltration assay for transiently expressing SAP11 and AtTCPs, as described by Wang and coworkers [14], by expressing the two proteins (SAP11 and TCP) from the same vector to ensure that the two proteins would be expressed in the same cells and at the same time point. The results showed that the AP_SAP11-like_PM19Δ40–56 cannot destabilize the AtTCP3 and 4 of class II compared to the wt protein (Figure 7). However, it destabilizes AtTCP6 of class I and TCP12 of class II in the same manner as the wt protein. Interestingly the wt protein cannot destabilize the AtTCP19 of class I, although it binds to AtTCP19 in Y2H analysis.

## 4. Discussion

The SAP11 effector protein of AY-WB phytoplasma is thought to play a major role in the infection mechanism. Its interaction with and destabilization of *A. thaliana* CIN-TCP transcription factors leads to a deep disturbance of plant biological processes, resulting in morphological changes and a down-regulation of LOX2 expression and JA synthesis, and an increase of insect vector reproduction [6]. Although AP_SAP11-like protein bears little resemblance at the amino acid level to that of AY-WP_SAP11, it was suggested that they may have some similar functions. Janik and co-workers proved interaction of AP_SAP11-like protein with some *M. domestica* TCP transcription factors, and hence suggested an involvement in the hormonal changes occurring in apple trees during AP infection [10]. In *N. benthamiana*, AP_SAP11-like protein destabilizes TCP transcription factors, suppresses JA response, and alters the aroma phenotype, which may play a role in the attraction of the insect vector, in addition to the morphology of transgenic plants [13].

Despite their similar effect on plant development and plant hormone biosynthesis, to date, no protein domains have been identified that match each other in both proteins (AP_SAP11-like protein and AY-WB_SAP11), leaving functions of different areas of the AP_SAP11-like protein on a hypothetical basis. In this work, we focused on elucidating the role of the 40–56 amino acid stretch of AP_SAP11-like_PM19 protein on the localization of the protein in plant cells, the phenotype development in transgenic *A. thaliana* lines, and the AtTCP-binding activity.

It was reported that NLS of AY-WB_SAP11 protein is not required for binding some AtTCPs [7]. However, deletion of the *N*-terminal area of AY-WB_SAP11 including the NLS disrupts its ability to destabilize some AtTCPs, which leads to mild symptoms in *A. thaliana* transgenic plant lines [7]. To investigate the role of the 40–56 amino acid stretch of AP_SAP11-like_PM19 during symptom development, a mutant excluding amino acids 40 to 56 was generated. The results show that the *A. thaliana* lines expressing AP_SAP11-like_PM19 show the typical phenotypes (crinkled leaves and siliques, and witches’ broom) as were reported for plants expressing AY-WB_SAP11 [6]. However, the plants expressing AP_SAP11-like_PM19Δ40–56 showed no crinkled leaves and siliques, although the witches’ broom phenotype remains. This is consistent with the findings for AY-WB and wheat blue dwarf phytoplasma [6,14]. The RT-PCR results support the finding that the partial loss of the crinkled leaves and siliques phenotype in the plants expressing AP_SAP11-like_PM19Δ40–56 is not due to the expression level of the transgene.

Unfortunately, due to limited efficiency and consistency, *Agrobacterium*-mediated transient transformation in Col-0 is not efficient [30,31,32,33]. Thus, nuclear localization was studied in *Agrobacterium*-mediated transient *N. benthamiana* leaves.

Because our protein localization results show that AP_SAP11-like_PM19 and the Δ40–56 mutant are distributed in the plant cells in the same manner, the effect of amino acids 40–56 on the protein localization can be excluded. Thus, the result suggests that this amino acid stretch is not responsible for the nuclear import of the protein. The same finding was reported for a SAP11-like effector, SWP1, isolated from wheat blue dwarf phytoplasma. It was shown that deletion of the C-terminal-predicted NLS of the protein did not affect the localization of the protein into the nucleus [14]. Therefore, the localization of the expressed protein is not the major cause for losing phenotypes in the transgenic plants expressing AP_SAP11-like_PM19Δ40–56.

The result of the Y2H screening, the interaction of AP_SAP11-like_PM19, and the Δ40–56 mutant with AtTCPs, indicate that the 40–56 amino acid stretch is not required for binding all AtTCPs but it is necessary for binding of AtTCPs 3, 4, 10, and 19.

TCP transcription factors have been intensively studied in recent years, showing that they regulate a variety of plant processes from plant development to defense responses. The functions of different AtTCPs and their role in biosynthetic processes have been reviewed in detail by Shutian Li [34]. The variety of their functions ranges from developmental processes and involvement in the clock oscillator to defense response [34].

We showed that the 40–56 amino acid stretch of AP_SAP11-like_PM19 is important for binding to class II AtTCPs 3 and 4, and losing its binding activity, resulting in AtTCP 3 and 4 not being destabilized in vivo. It should be noted that, in our results, AP_SAP11-like_PM19 strongly destabilized AtTCP3 and 4, whereas Chang and co-workers showed that the AP_SAP11-AT strain destabilizes AtTCP3 and 4 at low activity [23]. These different results could be due to the differences in the expression systems used. In our experiment, the two proteins were expressed from the same vector, and therefore the proteins were expressed in the same cells and at the same time point, which may have optimized the destabilization assay. Alternatively, a substitution of glutamic acid (E) in the TCP binding area of the AT strain by aspartic acid (D) of the PM19 strain (Figure 1a) may strengthen the TCP binding, which consequently leads to an increasing destabilization of the proteins. The three AtTCPs (3, 4, and 10) are closely related to one another [34], and belong to a group of five AtTCPs that are regulated by the microRNA miR319 [35]. Overexpression of miR319 leads to down-regulation of AtTCPs 2, 3, 4, 10, and 24 and a crinkled leaf phenotype [35]. Crinkled leaves and/or siliques have also been observed in other *A. thaliana* mutants when the members of this group of AtTCPs are down-regulated [36,37]. These findings, together with our Y2H screening results and TCP destabilizing assays with AtTCP3 and 4, suggest that the loss of binding and destabilizing activity with at least AtTCP 3 and 4 of AP_SAP11-like_PM19Δ40–56 are involved in the disappearance of the crinkled leaves and siliques phenotype in the transgenic plants.

However, the transgenic *A. thaliana* lines expressing AP_SAP11-like_PM19Δ40–56 still shows the witches’ broom phenotype and smaller rosettes. This could be due to the binding and destabilizing ability of AP_SAP11-like_PM19Δ40–56 of at least AtTCP12, which redundantly controls branch overgrowth [38,39,40]. The same result was obtained in transgenic plants expressing the deletion of the predicted NLS of SAP11-like effector, SWP1 [14].

The activity of TCPs is controlled by chromatin remodeling complexes BRM, TIE1, or SPL [41]. It was proposed that the TCP4 interacts with a BRM chromatin-remodeling complex to regulate leaf maturation and modification of the chromatin state of promoters of common targets such as ARR16 and ARR6. This provides a temporal cue to dampen Cytokinin responses, thus restricting morphogenetic programs that initially are active throughout leaf primordia and are later restricted to leaf margins (blastozones) [42]. TIE1 suppresses the activity of TCPs by acting as a bridge connecting corepressor TPL/TPRs with CIN-like TCPs during leaf development [43]. It was shown that overexpression of TIE1 resulted in excessive branches [44]. Thus, the reduction the TCPs destabilized by SAP11 could be a negative feedback to the expression level of BRM and TIE1, resulting in curled leaves and the witches’ bloom phenotype, respectively. Therefore, it would be of interest to study the impact of SAP11 on chromatin remodeling complexes in more detail.

Moreover, our result showed that AP_SAP11-like_PM19 can bind and destabilize some class I AtTCPs. Although these effects on the plant phenotype are not investigated, it demonstrates that AP_SAP11-like_PM19 bind to TCPs diversely and selectively. To date, it is still unclear how SAP11 binds and destabilizes the AtTCPs selectively; however the binding and destabilizing activities of AP_SAP11-like_PM19Δ40–56 on some AtTCPs (TCP12 and TCP6) suggest that this amino acid stretch is not required for binding and destabilizing of these AtTCPs, but it is necessary for binding at least to AtTCP3 and 4. However, it cannot be concluded that the binding of SAP11 to AtTCPs will lead in all cases to the destabilization of TCPs, because the binding of SAP11 to AtTCP19 does not cause the destabilization of this protein. Therefore, our results suggest that the requirements for the binding and destabilizing activity of AP_SAP11-like_PM19 depend on each TCP individually.

In higher plants, growth and development are achieved through genetically programmed, concerted processes that are controlled by intrinsic signaling pathways that respond to environmental signals [45]. The bacterial infection can avert the genetically programmed fate of meristem cells, thereby drastically altering the growth pattern of the host plant. SAP11 effectors exhibit the ability to enhance the proliferation of axillary meristems, which is responsible for the witches’ broom symptom. Although the molecular mechanisms SAP11-induced proliferation of axillary meristems remains unknown, it was shown that the potato purple top phytoplasma infected tomato plants show witches’ broom symptoms and the transcript levels of the tomato *WUS* ortholog, *LeWUS*, are decreased in the meristems of lateral shoot apices from phytoplasma-infected plants [45]. This finding is similar to the recessive *WUS* mutant Arabidopsis, which is evident in the apparent repeated initiation and outgrowth of vegetative meristem from the axils of previously produced leaves and the generation of a “stop-and-go”-like pattern of auxiliary bud growth, giving rise to a bushy appearance [46]. Because *WUS* expression positively regulates the SNF2 chromatin-remodeling ATPase SPLAYED (SYD), which is required for maintenance of the stable stem cell pool [47], it is possible that SAP11 may induce alterations of growth patterns by disturbing the transcriptional reprogramming of key meristem switching genes (such as WUS and SYD), possibly via TCPs, which are finely controlled by microRNA319 (miRNA319), chromatin remodeling complexes, and auxin homeostasis [41]. However, this speculation needs to be experimentally identified. In addition, the formation of leaves and the development of marginal outgrowths that form the final leaf shape are controlled by Auxin transport [48]. Thus, it would also be of interest to verify the auxin signaling response in the transgenic plants expressing SAP11-PM19.

## 5. Conclusions

Our results show that expressing AP_SAP11-like_PM19 in *A. thaliana* leads to a change of phenotype in transgenic plants (witches’ broom, crinkled leaves and siliques, and increase in number of primary stems). Moreover, our results strongly suggest that the nuclear localization of the AP_SAP11-like_PM19 protein is not due to the 40–56 amino acid stretch which has been predicted to be an NLS. In addition, this stretch is at least a part of the binding site with some AtTCPs, which are important in controlling the plant leaf morphology. Moreover, the AP_SAP11-like_PM19 can not only bind all AtTCPs of class II but also some of those of class I.

## Figures and Tables

**Table 1:** Y2H results of AP_SAP11-like_PM19 and AP_SAP11-like_PM19Δ40–56 with different AtTCPs.

AtTCP	Subclass	Interaction with AP_SAP11_like_ PM19	Interaction with AP_SAP11_like_ PM19Δ40–56
1	Class II	pos.	pos.
2	Class II	pos.	pos.
3	Class II	pos.	neg.
4	Class II	pos.	neg.
5	Class II	pos.	pos.
6	Class I	pos.	pos.
7	Class I	pos.	pos.
8	Class I	neg.	n.a.
9	Class I	pos.	pos.
10	Class II	pos.	neg.
11	Class I	neg.	n.a.
12	Class II	pos.	pos.
13	Class II	pos.	pos.
14	Class I	pos.	pos.
15	Class I	neg.	n.a.
16	Class I	neg.	n.a.
17	Class II	pos.	pos.
18	Class II	pos.	pos.
19	Class I	pos.	neg.
20	Class I	neg.	n.a.
21	Class I	pos.	pos.
22	Class I	neg.	n.a.
23	Class I	neg.	n.a.
24	Class II	pos.	pos.

pos. = Interaction of proteins in Y2H; neg. = No interaction of proteins in Y2H; n.a. not applicable.

## Data Availability

No new data were created or analyzed in this study. Data sharing is not applicable to this article.

## Supplementary Materials

The following are available online at https://www.mdpi.com/article/10.3390/microorganisms9081756/s1, Figure S1: Y2H results of AP_SAP11-like_PM19 and AP_SAP11-like_PM19Δ40–56 with different AtTCPs. Table S1: Gene-specific primer used for RT-qPCR. Table S2: Average Cq values, standard error, and maximal coefficient variation of Cq values within replicate samples of RT-qPCR.
