# Supplementary material for: The Effect of the Anticipated Nuclear Localization Sequence of ‘Candidatus Phytoplasma mali’ SAP11-like Protein on Localization of the Protein and Destabilization of TCP Transcription Factor
Source: Microorganisms. 2021 Aug 17;9(8):1756. doi: 10.3390/microorganisms9081756 (PMC8401217; doi:10.3390/microorganisms9081756)
Supplement: Supplementary file 1 [file microorganisms-09-01756-s001.zip › microorganisms-1315596-supplementary.pdf]

The Effect of the Anticipated Nuclear Localization Sequence of '*Candidatus* Phytoplasma mali' SAP11-Like Protein on Localization of the Protein and Destabilization of TCP Transcription Factor

Alisa Strohmayer, Timothy Schwarz, Mario Braun, Gabi Krczal, Kajohn Boonrod*

Supplementary Material

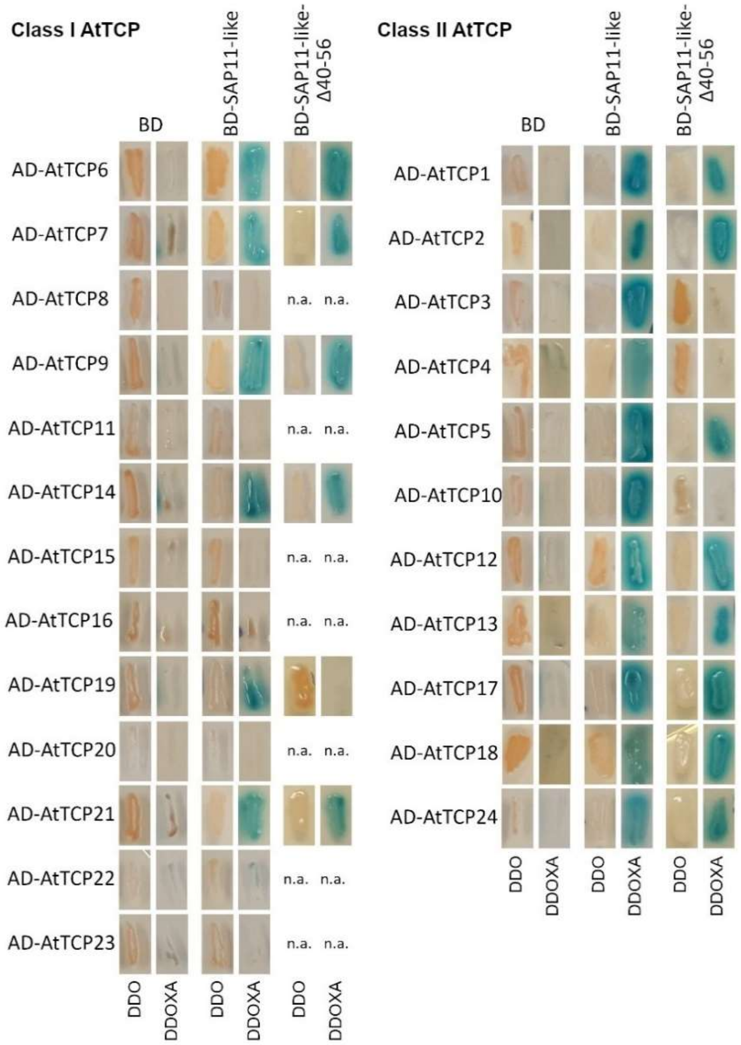

Figure S1: Y2H results of AP_SAP11-like_PM19 and AP_SAP11-like_PM19Δ40-56 with different AtTCPs. Y2H screens were performed using the binding domain fused to AP_SAP11-like_PM19 (BD-SAP11-like) or AP_SAP11-like_PM19Δ40-56 (BD-SAP11-like-Δ40-56) and the activation domain (AD) fused to different AtTCPs. For negative control only BD was used. All negative controls showed expected results (left column). AP_SAP11-like_PM19 binds to six AtTCP of class I and all of classII (middle column). Binding ability with AtTCP3, 4, 10 and 19 is lost, when aa 40 to 56 are deleted, while it remains with the other AtTCPs (right column). Results of these Y2H experiments are also summarized in Table 1.

Table S1: Gene-specific primer used for RT-qPCR.

Gene Name	Primer
AP_SAP11-LIKE_PM19	5′-CTTCCGGAAGAGGAG-3′
	5′-GATGAACTCCCACCTC-3′
PP2A	5′-TAACGTGGCCAAAATGATGC-3′
	5′-GTTCTCCACAACCGCTTGGT-3′
GAPDH	5′-TTGGTGACAACAGGTCAAGCA-3′
	5′-AAACTTGTCGCTCAATGCAATC-3′

Table S2: Average Cq values, standard error and maximal coefficient variation of Cq values within replicate samples of RT-qPCR

Transgenic plant	Gene name	Average Cq value	Standard error of Cq	Maximal coefficient variation of Cq (%)
	SAP11-like	17.93	0.23	2.60
SAP11-like #4	*GAPDH*	16.78	0.03	0.39
	PP2A	21.27	0.13	1.18
SAP11-like #2	*SAP11-like*	16.81	0.21	2.48
	GAPDH	16.74	0.05	0.63
	PP2A	20.93	0.19	1.83
SAP11-like #3	*SAP11-like*	17.44	0.16	1.88
	GAPDH	16.76	0.02	0.18
	PP2A	21.18	0.11	1.02
Δ40-56 #11	*SAP11-like*	17.32	0.43	4.91
	GAPDH	16.62	0.03	5.17
	PP2A	21.82	0.14	1.29
Δ40-56 #4	*SAP11-like*	17.84	0.46	0.31
	GAPDH	16.62	0.03	0.32
	PP2A	21.96	0.14	1.25
	SAP11-like	18.28	0.65	7.14
Δ40-56 #3	*GAPDH*	16.63	0.02	0.27
	PP2A	22.19	0.08	0.74

SAP11-like = AP_SAP11-like_PM19, Δ40-56 = AP_SAP11-like_PM19Δ40-56
